# The Chauvet 2025 meeting report: an update on current psychosocial research in vascularized composite allotransplantation

**DOI:** 10.3389/frtra.2026.1900898

**Published:** 2026-07-22

**Authors:** Helen Stark, Sheila G. Jowsey-Gregoire, Simon Talbot, David Leonard, Patrik Lassus, Hatem Amer, Vasyly Rohovyy, Emmanuel Morelon, Palmina Petruzzo, Maggie Bellew, Martin Kumnig

**Affiliations:** 1 Translational Research in Immunology Group, Nuffield Department of Surgical Sciences, University of Oxford, Oxford, United Kingdom; 2 Department of Psychiatry and Psychology, Mayo Clinic Jacksonville, Jacksonville, FL, United States; 3 Division of Plastic Surgery, Brigham and Women’s Hospital, Boston, MA, United States; 4 Department of Plastic & Hand Surgery, Leeds Teaching Hospital NHS Trust, Leeds, United Kingdom; 5 Department of Musculoskeletal and Plastic Surgery, University of Helsinki, Helsinki, Finland; 6 Transplant Center & Division of Nephrology and Hypertension, Mayo Clinic Rochester, Rochester, MN, United States; 7 Voice of VCA Patients, Innsbruck, Austria; 8 Department of Transplantation, Edouard Herriot Hospital, Lyon, France; 9 Department of Surgery, University of Cagliari, Cagliari, Italy; 10 Department of Plastic Surgery, St James’s University Hospital, Leeds, United Kingdom; 11 Department of Psychiatry, Psychotherapy, Psychosomatics and Medical Psychology, Center for Advanced Psychology in Plastic and Transplant Surgery, Medical University of Innsbruck, Innsbruck, Austria

**Keywords:** evaluation and follow-up, face transplantation, limb transplantation, outcomes research, quality of life, team science, vascularized composite tissue allotransplantation

## Abstract

Under the auspices of the International Society of Vascularized Composite Allotransplantation (ISVCA), a section of the Transplantation Society (TTS) that promotes and encourages research and training in the field of vascularized composite allotransplantation (VCA), the 4th Chauvet Workshop was convened in conjunction with the biennial meeting of the ISVCA from 27–29th June 2025 in Helsinki, Finland. The Chauvet workgroup recognizes the importance of psychological evaluation and management in VCA and works to provide an interdisciplinary platform for the development of psychosocial guidelines for the field. For the first time this workshop was conducted jointly with the ISVCA biennial congress, facilitating wider attendance, and discussions between different units and across disciplines. Three main areas were discussed; outcomes and lessons learned, qualitative research approaches and patient reported outcome measures (PROMS), and the role of team science in complex care models. We also heard an update from patient advocate Vasyly Rohovyy, on the progress being made to develop a VCA patient support and advocacy community. Here we summarize these discussions.

## Introduction

The 4th Chauvet Workshop was convened from 27–29th June 2025, in Helsinki, Finland. VCA presents a newer field within the transplantation community that unites many disciplines to achieve its primary goal: restoring patients’ dignity and function, enabling them to reach their maximum potential, and increasing their quality of life. 80 leading experts in the field (Supplementary Appendix 1), representing 23 teams took part in the workshop.

This manuscript is submitted as a Meeting Report. The conclusions summarized herein reflect intensive interdisciplinary discussions during the workshop and were refined through plenary discussion until consensus was achieved among the participating experts rather than by formal voting.

## Theme 1: outcomes, updates, and lessons learned from VCA transplant centers

Presentations from teams in Lyon, Boston and Helsinki reported on several years of follow up and allowed the opportunity to reflect on the factors that contribute to good outcomes. These can broadly be split into those relating to the patient and those relating to the environment and team undertaking the transplant.

Patient factors critical for success included low depression and anxiety scores, high levels of patient participation and adherence, resilience, good coping skills, a realistic expectation of outcome and strong family and/or social support (1, 2). All teams assessed these factors when considering patients for transplant, to understand their needs and optimize them prior to surgery.

It was also interesting to consider the role that national culture plays in the outcomes of VCA. In general Finnish culture promotes altruism, and organ donation is seen as morally good. There is respectful media coverage with no intrusion and little-to-no sensationalism, contributing to broad acceptance of VCA. VCA in Finland is offered through the Finnish health system as an extension of clinical practice like solid organ transplantation.

The experience of these centers was recognized by the teams who are developing laryngeal transplant programs in the US and has been used to inform their programs (3, 4). They noted that it is difficult to consent patients for a procedure with which you have little experience and few cases to demonstrate reliable outcomes, a topic that resonates with the shared purposeful decision making that Sheila Jowsey-Gregoire discussed in more detail below. It is also challenging to judge the success of the procedure, for example not all patients recover the ability for oral feeding but may recover speech. Involving patients in developing assessment and follow-up measurements for success prior to and post transplant will be crucial.

## Theme 2: qualitative research approaches and patient reported outcomes research

The consent process for VCA, especially for those with limited numbers such as laryngeal transplantation, is complex. Sheila Jowsey-Gregoire shared her team's work on purposeful shared decision making (5), a framework that was developed to facilitate the consent process and aid in decision making for patients and teams (6). This resulted from qualitative interviews with patients who had limb loss, transplant recipients, caregivers and providers. It can be applied not only to those awaiting hand transplantation, but to all forms of VCA.

The importance of measuring outcomes that are meaningful for patients was highlighted and forms the core of work being undertaken by the TORCH (transplantation outcomes research collaborative for the hand) group (7). They are developing a short subset of measures, based on the health-related quality of life domains identified as most important by upper limb loss patients and the health professionals involved in their care. These tools are necessary to allow monitoring of patients post-HAUL (hand and upper limb) transplantation.

In contrast to the US, HAUL transplantation in the UK is a centralized, NHS funded program. This cohort of patients have taken part in research focusing on exploring their lived experience of undergoing a HAUL-transplant in the UK. It will be of interest to see how their experience compares to patients who have received a similar transplant but delivered within a different healthcare system.

Dr Fay Bound Alberti challenged the audience about the future of face VCA, highlighting the need to develop collaborative frameworks for managing these patients, coupled with a long-term perspective on what constitutes a good outcome (8). She highlighted the conflicts that can arise in privatized healthcare systems that may contribute to poor experiences for VCA patients. The newly formed Clinical Organization Network for Standardization of Reconstructive Transplantation (CONSORT) recognizes these challenges and aims to address them in a collaborative manner. This has included a commissioned report by the National Academies of Science, Engineering, and Medicine (NAESM) on the state of VCA and the development of a consensus driven clinical trial protocol and network of clinical centers that will implement this protocol (9).

## Theme 3: complex care models, role of team science and collaborative models

VCA teams are an example of a complex care model, that can be analyzed using a team science approach (10, 11). They require intense collaboration across multidisciplinary teams. Having clear institutional values as a core set of principles to guide the goals of the team is a good starting point. By summarizing the mains results from his qualitative research on the role of team science and multidisciplinary collaborations in VCA, Martin Kumnig emphasized the benefits of learning from existing programs with established expertise. It was recommended that VCA programs are developed in units which already have experience with solid organ transplantation. Key requirements for developing an effective team were regular meetings, with well-planned communication and leadership plans, these help to build trust and manage any conflict at an early stage. In the pre-transplantation period using standardized evaluations and consent processes were felt to be of value. Finally post-transplantation there is a need for ongoing rehabilitation and education, to aid adherence to treatment protocols. Those teams which utilized these strategies demonstrated improved team learning, patient selection and good transplant outcomes. The importance of securing sustainable financial support from the institution was noted (12).

## Theme 4: VCA patient community

Vasyly Rohovyy, who underwent bilateral hand and upper limb transplantation at the Medical University Innsbruck, Austria in 2006, presented the ‘Voice of VCA Patients’ a concept of creating an international VCA patient community, in collaboration with and moderated by leading experts in the field. The aim is to provide a platform where patient who have a VCA or are considering one, may interact with each together. Beyond the important exchange of information, this initiative raises awareness about VCA-related issues, e.g., post-transplant life, helping in the decision-making process and risk-benefit considerations, patient mentoring and advocacy. More information can be found using the url: https://worldofvca.com.

## Conclusions and future directions

The Chauvet meeting brought together a cross-disciplinary group of professionals, with the overarching goal of improving patient outcomes. The psychosocial impact of VCA is well recognized. There are now several groups focusing their efforts on developing outcome measures that will allow us to assess the impact on the clinical outcomes. Furthermore, the discipline of team science is being used to optimize programs which deliver VCA. The voice of patients is being incorporated, and amplified, and careful listening will help direct research priorities. Participants agreed that future priorities include strengthening multicenter collaborations, harmonizing psychosocial assessment and patient-reported outcome measures, supporting collaborative research networks, and working toward consensus recommendations and minimum standards as evidence matures.

## Data Availability

The raw data supporting the conclusions of this article will be made available by the authors, without undue reservation.
