# Supplementary material for: The Chauvet 2025 meeting report: an update on current psychosocial research in vascularized composite allotransplantation
Source: Front Transplant. 2026 Jul 22;5:1900898. doi: 10.3389/frtra.2026.1900898 (PMC13438373; doi:10.3389/frtra.2026.1900898)
Supplement: Supplementary file 1 [file Supplementaryfile1.pdf]

The Supplementary Material for this article can be found online

Listing of All Participants and Their Associated Transplant Programs

First name	Last name	Organization	Country
Hatem	Amer	Mayo Clinic	United States
Benita	Antas	HUS	Finland
Jun	Araki	Shizuoka Cancer Center	Japan
Isabel	Arenas Hoyos	Inselspital	Switzerland
Petri	Arvilommi	HUS	Finland
Veera	Astikainen	HUS yhtymä tukielin- ja plastiikkakirurgia	Finland
Juan Pedro	Barret Nerin	HOSPITAL UNIVERSITARIO VALL D'HEBRON	Spain
Maggie	Bellew	Leeds Teaching Hospitals NHS Trust	United Kingdom
Oriol	Bermejo	BIOCABLAN S.L.	Spain
Fay	Bound Alberti	King's College London	United Kingdom
Grainne	Bourke	Leeds teaching hospitals trust	United Kingdom
Joanna	Burdon	Leeds Teaching Hospitals NHS Trust	United Kingdom
Magda Patricia	Butron Gandarillas	INCMNSZ	Mexico
Curtis	Cetrulo	Cedars-Sinai Medical Center	United States
Harini	Chakkera	Mayo Clinic	United States
Yu-Han	Chen	Kaohsiung Medical University Chung-Ho Memorial Hospital	Taiwan
Ruth	Cooper	National Academies of Sciences, Engineering, and Medicine	United States
Carl	Cramer	Mayo Clinic	United States
Efrat	Daglan	Leeds teaching hospital	United Kingdom

Michael	Davis	MD3 Multidimensional Medical Consulting	United States
Raghuveer Reddy	Dudipala	Continental hospitals, Hyderabad	India
Jérôme	Duisit	Iris South Hospitals Brussels	Belgium
Cagdas	Duru	University Health Network, Toronto	Canada
Christophe	Gaillard	Hospices Civils de Lyon	France
Aram	GAZARIAN	.	France
Bruce	Gelb	NYU Langone Health	United States
Karely	Gonzalez Chaparro	Laboratorio de Microcirugía "Dr Francisco Javier Huerta Rivadeneyra"	Mexico
Nina	Hadzimustafic	McGill University	Canada
Siba	Haykal	Yale University	United States
Julie	Heimbach	Mayo Clinic Rochester	United States
Pauliina	Homsy	Helsinki University Hospital	Finland
Martin	Iglesias Morales	Orange Medical/DioMed Hospital	Mexico
Naim	Issa	Mayo Clinic	United States
Vinay	Itte	Leeds Teaching Hospitals NHS Trust	United Kingdom
Subramania	Iyer	Amrita Institute of Medical Sciences	India
Nicole	James	Oxford University Hospitals NHS Foundation Trust	United Kingdom
Jelena	Janjic	Duquesne University	United States
Sheila	Jowsey-Gregoire	Mayo Clinic	United States
Olena	Kalchenko	The Voice of VCA Patient	Austria
Andrea	Kattah	Mayo Clinic	United States
Christina	Kaufman	University of Louisville	United States

Patricia	Kehn	NIH	United States
Brogan	Kerr	Leeds Teaching Hospitals NHS Trust & University of Leeds	United Kingdom
Emma-Lotta	Kiukas	EKHVA	Finland
Martin	Kumnig	Medical University of Innsbruck	Austria
Yur-Ren	Kuo	Kaohsiung Medical University Chung-Ho Memorial Hospital	Taiwan
Josef	Kurtz	Emmanuel College	United States
Patrik	Lassus	Helsinki University Hospital	Finland
Chia-Chun	Lee	Kaohsiung Medical University Chung-Ho Memorial Hospital	Taiwan
David	Leonard	Leeds Teaching Hospitals NHS Trust	United Kingdom
L Scott	Levin	Department of Orthopaedic Surgery 10th Floor	United States
Andrew	Lindford	Helsinki University Hospital	Finland
David	Lott	Mayo Clinic Phoenix	United States
Alexia	Lozada Estrada	Instituto Mexicano del Seguro Social	Mexico
Elise	Lupon	CHU de Nice	France
Rachel	Mandela	Leeds Teaching Hospitals	United Kingdom
Samir	Mardini	Mayo Clinic	United States
Jari	Mauno	Helsinki University Hospital	Finland
Christine	McAndrew	Penn Medicine	United States
Emmanuel	Morelon	Hôpital Edouard Herriot	France
Elisa	Moreno	Los Angeles Dept. Mental Health	United States
Girish	Mour	Mayo Clinic	United States
Antti	Mäkitie	University of Helsinki	Finland

Dimitris	Nakos	Melina Nakos Foundation	United States
Noela	Nakos	Melina Nakos Foundation	United States
Palmina	Petruzzo	Dept of Surgery, Cagliari University	Italy
Georgios	Pierides	Helsinki University Central Hospital	Finland
Raymund	Razonable	Mayo Clinic	United States
Claudio Francisco	Reyes Montero	Hospital Central del Estado de Chihuahua / TLALPAN Team	Mexico
Robert	Rieben	University of Bern, DBMR	Switzerland
Vasyly	Rohovyy	The Voice of VCA Patients	Austria
Terry	Schneekloth	Mayo Clinic	United States
Mohit	Sharma	Amrita Hospital	India
Richard	Sharp	Mayo Clinic	United States
Maria	Siemionow	University of Illinois-Chicago	United States
Helen	Stark	University of Oxford	United Kingdom
Viola	Stoegner	Yale School of Medicine	United States
Simon	Talbot	Brigham and Women's Hospital	United States
Sarah	Taplin	Leeds Teaching Hospitals NHS Trust	United Kingdom
Sylvie	Testelin	University Hospital Amiens- Picardie	France
Suzanne	Thomson	Leeds Teaching Hospital Trust	United Kingdom
David	Tulsky	University of Delaware	United States
